# Supplementary material for: Impact of statins as immune-modulatory agents on inflammatory markers in adults with chronic diseases: A systematic review and meta-analysis
Source: PLoS One. 2025 May 29;20(5):e0323749. doi: 10.1371/journal.pone.0323749 (PMC12121830; doi:10.1371/journal.pone.0323749)
Supplement: S6 Table — (DOCX) [file pone.0323749.s006.docx]

**S6 Table. Overall analysis of randomized controlled trials.**

| **Groups** | **Outcomes** | **Criteria** | **Studies (n)** | **Intervention (n)** | **Control (n)** | **Mean Differences (95 CI)** | **I^2^ (95 CI)** | **P-value** | **P-Egger's test** |
| --- | --- | --- | --- | --- | --- | --- | --- | --- | --- |
| **Overall** |  |  |  |  |  |  |  |  |  |
|  | LDL-C | Overall | 30 | 14240 | 13786 | -0.98 [-1.17; -0.78] | 98.1 [97.7; 98.3] | <0.001 | 0.022 |
|  |  | Atorvastatin | 16 | 1992 | 1995 | -1.10 [-1.31; -0.88] | 94.1 [91.8; 95.7] | <0.001 | 0.458 |
|  |  | Simvastatin | 7 | 2534 | 2502 | -0.78 [-1.16; -0.39] | 98.8 [98.4; 99.1] | <0.001 | 0.044 |
|  |  | Pravastatin | 1 | 30 | 30 | -0.49 [-0.79; -0.19] | NC | NC | NC |
|  |  | Rosuvastatin | 4 | 9660 | 9239 | -1.48 [-1.86; -1.10] | 97.9 [96.6; 98.8] | <0.001 | 0.551 |
|  |  | Fluvastatin | 2 | 24 | 20 | -0.06 [-0.23; 0.10] | 0.0 [0.0-0.0] | 0.879 | NC |
|  | HDL | Overall | 26 | 13455 | 13423 | 0.06 [0.03; 0.09] | 63.5 [44.8; 75.8] | <0.001 | 0.967 |
|  |  | Atorvastatin | 15 | 1959 | 1963 | 0.06 [ 0.02; 0.10] | 65.0 [39.5; 79.8] | <0.001 | 0.362 |
|  |  | Pravastatin | 1 | 30 | 30 | 0.02 [-0.12; 0.16] | NC | NC | NC |
|  |  | Simvastatin | 5 | 2484 | 2453 | 0.08 [ 0.01; 0.14] | 74.3 [41.5; 88.7] | 0.002 | 0.492 |
|  |  | Rosuvastatin | 3 | 8958 | 8957 | 0.10 [0.01; 0.19] | 30.2 [0.0; 92.7] | 0.238 | 0.054 |
|  |  | Fluvastatin | 2 | 24 | 20 | 0.00 [-0.07; 0.08] | 0.0 [0.0; 0.0] | 0.423 | NC |
|  | TG | Overall | 27 | 13428 | 13395 | -0.20 [-0.29; -0.11] | 91.5 [88.8; 93.6] | <0.001 | 0.608 |
|  |  | Atorvastatin | 16 | 1946 | 1949 | -0.24 [-0.35; -0.12] | 82.5 [72.7; 88.8] | <0.001 | 0.538 |
|  |  | Simvastatin | 6 | 2506 | 2474 | -0.25 [-0.41; -0.08] | 74.8 [42.8; 88.9] | 0.001 | 0.068 |
|  |  | Pravastatin | 1 | 30 | 30 | -0.01 [-0.51; 0.48] | NC | NC | NC |
|  |  | Rosuvastatin | 2 | 8922 | 8922 | -0.23 [-0.25; -0.21] | 0.0 [0.0; 0.0] | 0.750 | NC |
|  |  | Fluvastatin | 2 | 24 | 20 | 0.08 [-0.16; 0.32] | 93.7 [79.9; 98.1] | <0.001 | NC |
|  | TC | Overall | 27 | 4583 | 4550 | -1.01 [-1.24; -0.78] | 97.2 [96.5; 97.7] | <0.001 | 0.010 |
|  |  | Atorvastatin | 15 | 1938 | 1942 | -1.24 [-1.55; -0.93] | 95.7 [94.1; 96.8] | <0.001 | 0.580 |
|  |  | Simvastatin | 7 | 2534 | 2502 | -0.88 [-1.29; -0.46] | 97.0 [95.5; 98.0] | <0.001 | 0.003 |
|  |  | Pravastatin | 1 | 30 | 30 | -0.46 [-0.86; -0.05] | NC | NC | NC |
|  |  | Rosuvastatin | 2 | 57 | 56 | -1.04 [-1.60; -0.49] | 61.9 [0.0; 91.2] | 0.105 | NC |
|  |  | Fluvastatin | 2 | 24 | 20 | -0.13 [-0.35; 0.09] | 0.0 [0.0; 0.0] | 0.787 | NC |

n= total numbers; NC= Not Computable; LDL-C= Low-density lipoprotein-cholesterol; HDL= High-density lipoprotein; TG= Triglycerides; TC= Total cholesterol.
