# Supplementary material for: Impact of statins as immune-modulatory agents on inflammatory markers in adults with chronic diseases: A systematic review and meta-analysis
Source: PLoS One. 2025 May 29;20(5):e0323749. doi: 10.1371/journal.pone.0323749 (PMC12121830; doi:10.1371/journal.pone.0323749)
Supplement: S7 Table — (DOCX) [file pone.0323749.s007.docx]

**S7 Table. Analysis of randomized controlled trials stratified based on treatment duration.**

| **Groups** | **Outcomes** | | **Criteria** | **Studies (n)** | **Intervention (n)** | **Control (n)** | **Mean Differences (95 CI)** | **I^2^ (95 CI)** | **P-value** | **P-Egger test** |
| --- | --- | --- | --- | --- | --- | --- | --- | --- | --- | --- |
| **Treatment duration** | | **>4 months** |  |  |  |  |  |  |  |  |
|  | |  |  |  |  |  |  |  |  |  |
|  | | LDL-C | Overall | 12 | 11477 | 11051 | -0.92 [-1.24; -0.60] | 98.6 [98.2; 98.9] | <0.001 | 0.113 |
|  | |  | Atorvastatin | 5 | 1633 | 1628 | -0.79 [-1.10; -0.48] | 77.7 [46.2; 90.7] | 0.001 | 0.939 |
|  | |  | Simvastatin | 3 | 193 | 195 | -0.70 [-1.14; -0.27] | 88.7 [69.0; 95.9] | <0.001 | 0.345 |
|  | |  | Rosuvastatin | 3 | 9639 | 9218 | -1.61 [-1.96; -1.25] | 98.6 [97.6; 99.2] | <0.001 | 0.526 |
|  | |  | Fluvastatin | 1 | 12 | 10 | -0.05 [-0.26; 0.16] | NC | NC | NC |
|  | | HDL | Overall | 11 | 10775 | 10769 | 0.08 [0.04; 0.11] | 58.0 [18.0; 78.5] | 0.008 | 0.779 |
|  | |  | Atorvastatin | 5 | 1633 | 1628 | 0.11 [ 0.09; 0.13] | 0.0 [0.0; 79.2] | 0.751 | 0.336 |
|  | |  | Simvastatin | 3 | 193 | 195 | 0.06 [-0.03; 0.15] | 5.0 [0.0; 90.1] | 0.349 | 0.759 |
|  | |  | Fluvastatin | 1 | 12 | 10 | 0.09 [-0.02; 0.20] | 41.4 | 0.192 | NC |
|  | |  | Rosuvastatin | 2 | 8937 | 8936 | -0.03 [-0.14; 0.08] | NC | NC | NC |
|  | | TG | Overall | 11 | 10755 | 10751 | -0.14 [-0.25; -0.02] | 92.7 [88.9; 95.2] | <0.001 | 0.341 |
|  | |  | Atorvastatin | 6 | 1649 | 1645 | -0.15 [-0.24; -0.05] | 42.0 [0.0; 77.0] | 0.125 | 0.339 |
|  | |  | Simvastatin | 3 | 193 | 195 | -0.17 [-0.33; -0.01] | 0.0% [0.0%; 89.6%] | 0.402 | 0.996 |
|  | |  | Rosuvastatin | 1 | 8901 | 8901 | -0.23 [-0.25; -0.21] | NC | NC | NC |
|  | |  | Fluvastatin | 1 | 12 | 10 | 0.20 [ 0.13; 0.28] | NC | NC | NC |
|  | | TC | Overall | 9 | 1820 | 1815 | -0.74 [-1.02; -0.45] | 82.9 [69.0; 90.6] | <0.001 | 0.966 |
|  | |  | Atorvastatin | 4 | 1579 | 1575 | -0.70 [-1.06; -0.35] | 67.0 [3.8; 88.7] | 0.028 | 0.912 |
|  | |  | Simvastatin | 3 | 193 | 195 | -0.83 [-1.28; -0.37] | 81.6 [43.0; 94.1] | 0.004 | 0.512 |
|  | |  | Rosuvastatin | 1 | 36 | 35 | -1.30 [-1.72; -0.88] | NC | NC | NC |
|  | |  | Fluvastatin | 1 | 12 | 10 | -0.10 [-0.40; 0.20] | NC | NC | NC |
| **Treatment duration** | | **3-4 months** |  |  |  |  |  |  |  |  |
|  | | LDL-C | Overall | 18 | 2763 | 2735 | -1.02 [-1.26; -0.77] | 97.4 [96.7; 98.0] | <0.001 | 0.016 |
|  | |  | Atorvastatin | 11 | 359 | 367 | -1.24 [-1.48; -1.00] | 89.0 [82.3; 93.1] | <0.001 | 0.323 |
|  | |  | Simvastatin | 4 | 2341 | 2307 | -0.83 [-1.47; -0.20] | 99.2 [98.9; 99.5] | <0.001 | 0.288 |
|  | |  | Pravastatin | 1 | 30 | 30 | -0.49 [-0.78; -0.19] | NC | NC | NC |
|  | |  | Rosuvastatin | 1 | 21 | 21 | -0.91 [-1.50; -0.32] | NC | NC | NC |
|  | |  | Fluvastatin | 1 | 12 | 10 | -0.08 [-0.34; 0.18] | NC | NC | NC |
|  | | HDL | Overall | 15 | 2680 | 2654 | 0.05 [0.01; 0.09] | 57.1 [25.1; 75.4] | 0.003 | 0.782 |
|  | |  | Atorvastatin | 10 | 326 | 335 | 0.03 [-0.01; 0.05] | 32.2 [0.0; 67.7] | 0.150 | 0.966 |
|  | |  | Simvastatin | 2 | 2291 | 2258 | 0.08 [-0.02; 0.19] | 88.4 [67.9; 95.8] | <0.001 | 0.628 |
|  | |  | Pravastatin | 1 | 30 | 30 | 0.02 [-0.13; 0.17] | NC | NC | NC |
|  | |  | Rosuvastatin | 1 | 21 | 21 | 0.16 [-0.02; 0.34] | NC | NC | NC |
|  | |  | Fluvastatin | 1 | 12 | 10 | 0.03 [-0.07; 0.13] | NC | NC | NC |
|  | | TG | Overall | 16 | 2673 | 2644 | -0.25 [-0.36; -0.13] | 87.7 [81.6; 91.7] | <0.001 | 0.258 |
|  | |  | Atorvastatin | 10 | 297 | 304 | -0.27 [-0.43; -0.10] | 85.3 [74.8; 91.5] | <0.001 | 0.788 |
|  | |  | Simvastatin | 3 | 2313 | 2279 | -0.30 [-0.57; -0.02] | 79.1 [33.4; 93.5] | 0.008 | 0.516 |
|  | |  | Pravastatin | 1 | 30 | 30 | -0.01 [-0.50; 0.48] | NC | NC | NC |
|  | |  | Rosuvastatin | 1 | 21 | 21 | -0.28 [-0.59; 0.03] | NC | NC | NC |
|  | |  | Fluvastatin | 1 | 12 | 10 | -0.05 [-0.14; 0.05] | NC | NC | NC |
|  | | TC | Overall | 18 | 2763 | 2735 | -1.15 [-1.46; -0.84] | 95.8 [94.5; 96.8] | <0.001 | 0.008 |
|  | |  | Atorvastatin | 11 | 359 | 367 | -1.44 [-1.78; -1.10] | 92.6 [88.8; 95.2] | <0.001 | 0.488 |
|  | |  | Simvastatin | 4 | 2341 | 2307 | -0.91 [-1.60; -0.21] | 97.6 [95.9; 98.6] | <0.001 | 0.101 |
|  | |  | Pravastatin | 1 | 30 | 30 | -0.46 [-0.86; -0.05] | NC | NC | NC |
|  | |  | Rosuvastatin | 1 | 21 | 21 | -0.73 [-1.28; -0.18] | NC | NC | NC |
|  | |  | Fluvastatin | 1 | 12 | 10 | -0.16 [-0.48; 0.16] | NC | NC | NC |

n= Total numbers; NC= Not computable; IL-6= Interleukin 6; TNF-α= Tumor necrosis factor alpha; CRP= C-reactive protein; hs-CRP= High-sensitivity C-reactive protein; LDL-C= Low-density lipoprotein-cholesterol; HDL= High-density lipoprotein; TG= Triglycerides; TC= Total cholesterol
