# Supplementary material for: Impact of statins as immune-modulatory agents on inflammatory markers in adults with chronic diseases: A systematic review and meta-analysis
Source: PLoS One. 2025 May 29;20(5):e0323749. doi: 10.1371/journal.pone.0323749 (PMC12121830; doi:10.1371/journal.pone.0323749)
Supplement: Attachment — Submitted filename: Response to Reviewers.docx [file pone.0323749.s014.docx]

**Response to Reviewers**

The reviewers have found merit in this article, however, they also found several major flaws within the manuscript that need further clarification and revision. Major points that need to be clarified:

1) Introduction: please make the introduction section not too long. Focus on the background to justify this study, literature gaps, and purpose of this study.

**Answer:** We thank the reviewer for this suggestion. We have revised and shortened the introduction to focus on providing background justification for the study, addressing literature gaps, and clearly stating the study's purpose as follows:

“Statins are lipid-lowering drugs that have been prescribed to people at high risk of cardiovascular disease (CVD) since 1987 [1]. Statins lower cholesterol biosynthesis by inhibiting 3-hydroxy-3-methylglutaryl coenzyme A (HMG-CoA) reductase [2, 3]. This inhibition prevents the conversion of HMG-CoA into mevalonate, a pivotal substrate essential for cholesterol biosynthesis [4]. Despite their cardiovascular risk reduction properties, statins have shown to possess cholesterol-independent pleiotropic effects, including anti-inflammatory, anti-thrombotic, anti-oxidative, neuroprotective, anti-proliferative, plaque-stabilizing, and endothelial dysfunction-improving effects [5, 6]. These non-lipid pleiotropic effects are primarily mediated via statins-mediated inhibition of protein prenylation, a multistep enzymatic process in the mevalonate pathway that regulates protein-protein interaction and cell membrane protein anchoring [7]. A growing interest in drug repurposing has prompted investigations into statins as adjunctive host-directed therapies to reduce inflammation in various infectious and non-infectious inflammatory diseases [8]. This highlights the need for additional meta-analysis studies which may provide guidance and insight for future clinical trials on selecting the most potent statin based on inflammation type. While randomized control trials (RCTs), cohort studies, and non-randomized intervention studies have collectively assessed the effectiveness of different statins in mitigating inflammation across various diseases [9-12], significant gaps remain. Specifically, the comparative potency and optimal duration of different statins as anti-inflammatory agents are poorly elucidated. The purpose of this meta-analysis of randomized controlled trials is to evaluate the anti-inflammatory effects of statin therapy in patients with chronic diseases. Specifically, this study aims to identify which statins are most effective and determine the optimal duration of treatment for maximizing their anti-inflammatory benefits. The outcomes of this meta-analysis will feed into evidence-based protocols for statin therapy, addressing lipid abnormalities and mitigating inflammatory processes in disease progression. If proven effective, clinicians may consider the varying efficacy of statins in modulating inflammation in adults with chronic diseases.”Also see lines 70-119 in the revised manuscript.

2) Methods: please explain why this manuscript has several differences with the protocol.

**Answer**: We explained these differences in lines 151 -158 in the revised manuscript as follows:

“The inclusion criteria reported in the study protocol encompassed peer-reviewed articles published in English between 1999 and 2019. However, due to the enormous number of identified studies and delays in data extraction, analysis, and reporting, the inclusion criteria were adjusted to include studies published between 1st January 1999 and 31st December 2023. Studies were sourced from the PubMed, Web of Science, Scopus, and Cochrane databases. The literature search included randomized controlled trials (RCTs) and due to a significant number of non-randomized studies (NRS), these were included as a descriptive analysis”

3) Discussion: please elaborate more the findings from this study and be more analytical to explain those results.

**Answer**: We thank the reviewer for this suggestion. We have now provided a more analytical explanation of our results in the discussion, lines 461-634 in the revised manuscript.

“This systematic review and meta-analysis provide a comprehensive assessment of the effectiveness of statins in mitigating systemic inflammation and altering lipid profiles during chronic illnesses, with statin administration lasting 3 months or longer. This study aimed to shed light on the potential anti-inflammatory benefits of statin administration by encompassing a wide array of chronic diseases such as CVD, COPD, schizophrenia, and chronic renal failure. Through meticulous screening of literature across four distinct databases - PubMed, Web of Science, Scopus, and Cochrane - 27 RCT articles met the initial eligibility criteria. The findings from this meta-analysis highlight the significant impact of statin therapy in reducing systemic inflammatory markers, including IL-6, TNF-α, and CRP, across various chronic diseases. Analyzing data from multiple randomized controlled trials (RCTs), the study observed a consistent and statistically significant reduction in IL-6 (MD = -0.24 ng/dL), TNF-α (MD = -0.74 ng/dL), and CRP (MD = -1.58 mg/L) among participants receiving statins compared to those on placebo. Atorvastatin (20 mg) emerged as particularly effective in reducing IL-6 (MD = -5.39 ng/dL) and TNF-α (MD = -3.32 ng/dL), highlighting its potency in modulating these inflammatory markers (Table 1). Additionally, extended statin administration beyond four months demonstrated heightened efficacy in lowering IL-6 and TNF-α levels (Fig 3A-B), suggesting a potential temporal relationship between treatment duration and anti-inflammatory impact. However, data supporting the heightened efficacy for lowering TNF-α levels primarily originates from a single study (Fig 3B). IL-6, evaluated across seven RCTs involving 483 participants, and TNF-α, assessed in six RCTs involving 343 participants, both demonstrated substantial heterogeneity (IL-6: I² = 98.3%; TNF-α: I² = 98.8%), indicating variability in study outcomes despite overall significant reductions. Fifteen studies were included in CRP analysis with overall heterogeneity of I2=86.5% suggestive of high variety in included studies (Fig 2C and Table 1). The observed heterogeneity suggests that factors such as participant demographics, differences in study populations, baseline disease severity, treatment durations, and statin dosage might influence treatment outcomes. Notably, CRP levels were also reduced by statin treatment, especially by fluvastatin followed by rosuvastatin (Table 1). It's noteworthy that CRP, a non-specific marker of inflammation, is associated with CVD pathogenesis [46]. Data generated in this meta-analysis contrasts with those of the study conducted by Balk et al., 2003, where fluvastatin was observed to have the least impact on CRP reduction compared to other statins such as atorvastatin, simvastatin, and pravastatin [47]. However, it's important to acknowledge that this discrepancy in results could be attributed to the limited number of available studies that evaluated the efficacy of fluvastatin specifically in the context of chronic illness. While the existing evidence supports that statin treatment lowers systemic inflammatory markers levels, additional RCTs are still needed to delve deeper into the effects of statins on various inflammatory markers in chronic illnesses. The ultimate objective of these trials would be to establish a comprehensive understanding of how statin treatment influences a range of inflammatory markers and whether these effects translate into tangible improvements in disease-specific clinical outcomes.

Thirty studies encompassing over 28,000 participants demonstrated that statin therapy significantly reduced LDL-C levels (MD = -0.98 mmol/L), with substantial heterogeneity (I² = 98.1%). Rosuvastatin emerged as the most effective LDL-C reducer (MD = -1.48 mmol/L), followed by atorvastatin (MD = -1.10 mmol/L) and simvastatin (MD = -0.78 mmol/L). This efficacy trend has been previously reported by Schachter M. et al., 2005, which showed that rosuvastatin led to a 63% reduction in LDL-C, followed by atorvastatin with 50% and simvastatin with 41% [48]. Extended treatment duration (> 4 months) with rosuvastatin showed the greatest potency in lowering LDL-C concentrations (Fig 4B, S7 Table). These findings underscore the differential efficacy of statins in reducing LDL-C, reflecting their varying pharmacokinetic profiles and potency in inhibiting cholesterol synthesis via the mevalonate pathway. Twenty-seven studies involving over 26,800 participants demonstrated a significant reduction in TG levels with statin therapy (MD = -0.20 mmol/L, I² = 91.5%). Similarly, statins significantly decreased TC levels across twenty-seven studies (MD = -1.01 mmol/L, I² = 97.2%). Atorvastatin exhibited the most potent effect on TC reduction (MD = -1.24 mmol/L), followed by rosuvastatin (MD = -1.04 mmol/L) and simvastatin (MD = -0.88 mmol/L). The variability in efficacy among statins for TG and TC reductions may stem from differences in lipid metabolism pathways targeted by each statin. The high heterogeneity observed across lipid outcomes underscores the importance of considering factors such as baseline lipid levels, statin dosage, and study duration when interpreting results. Heterogeneity may also reflect variations in participant characteristics and study methodologies. The stratification by treatment duration highlights the potential for longer-term statin therapy, particularly with rosuvastatin, to achieve more pronounced lipid-lowering effects. These findings have significant implications for clinical practice, suggesting that statins, particularly rosuvastatin and atorvastatin, are effective in managing dyslipidemia across diverse patient populations. Tailoring statin therapy based on individual lipid profiles and treatment duration may optimize lipid control and reduce cardiovascular risk. Clinicians should consider the differential effects of statins on LDL-C, TG, and TC when selecting treatment regimens for patients with specific lipid management goals.

An important limitation of this meta-analysis is that the lipid-lowering effect sizes of the various statins are derived from highly heterogeneous studies with variable baseline lipids and data on LDL-C and TC changes were not available for all statins. These results should thus not be interpreted as direct comparisons of statin potency. In this study, we used random-effects models to account for variability among studies and provide more conservative estimates of the overall effect. Also, sensitivity analyses were performed to assess the robustness of the findings by excluding studies with skewed evidence, as shown in S11 Table. Despite these efforts, high heterogeneity remains a limitation that impacts the generalizability of the meta-analytic conclusion. To address publication bias, we employed comprehensive literature search strategies, including backward citation searching and trial registries. Additionally, we used statistical methods such as funnel plots and Egger’s test to detect and adjust for potential bias. However, these methods have limitations, and the possibility of residual bias cannot be entirely ruled out.

In conclusion, this meta-analysis provides robust evidence supporting the anti-inflammatory effects of statins across various chronic diseases. Atorvastatin, fluvastatin, and rosuvastatin emerged as particularly effective in reducing IL-6, TNF-α, and CRP levels, respectively. The greater anti-inflammatory efficacy of rosuvastatin and atorvastatin is primarily attributed to their minimal first-pass effect and relatively slow hepatic elimination. In contrast, other statins such as pravastatin undergo extensive first-pass metabolism and are rapidly inactivated by the liver before reaching systemic circulation. As a result, atorvastatin and rosuvastatin have longer elimination half-lives of 14 and 19 hours, respectively, while pravastatin's elimination half-life is only 2 hours. These findings have significant clinical implications for the management of chronic diseases characterized by inflammation, such as cardiovascular disease and chronic renal failure. Incorporating statin therapy into treatment protocols not only addresses lipid abnormalities but also potentially mitigates inflammatory processes implicated in disease progression. Clinicians may consider the differential efficacy of statins in inflammation modulation when tailoring treatment strategies for individual patients.”

**Reviewer #2:** This is a well-written meta-analysis to assess the impact of statins as immune-modulatory agents on inflammatory markers. I have the following comments:

**Answer:** We thank the reviewer for this overall appreciation.

1. Please report if the high risk of bias in studies is an exclusion criterion in the methods or not.

**Answer**: The high risk of bias is not an exclusion criterion. We assessed the risk of bias only in the included studies classified as high methodological quality based on JADAD scale scoring, while those with low methodological quality were excluded from the meta-analysis. (lines 170-172 in the revised manuscript). The assessment of methodological quality and risk of bias is presented in the revised manuscript, lines 195-224.

2. Add a table to assess the quality of evidence of each outcome using the GRADE approach.

**Answer:** We thank the reviewer for this suggestion. We now assessed the certainty of the evidence for inflammatory biomarker changes using Grading of Recommendations, Assessment, Development, and Evaluations (GRADE) approach. Accordingly, we added GRADE assessment to Table 1 and Table 2 and have added the following texts to the revised manuscript, lines 371-380.

“We found no high-quality evidence to answer our research question. The certainty of evidence was generally low for IL-6 and TNF-α, and moderate for CRP and hs-CRP outcomes. Factors contributing to the reduction in certainty were inconsistency and imprecision (Table 1). For outcomes with treatment duration longer than 4 months, the certainty of the evidence was categorised as low for IL-6 and TNF-α, and moderate for CRP and hs-CRP (Table 2). Whereas the certainty of evidence was very low for IL-6 and TNF-α, and was low for CRP and hs-CRP with the treatment duration 3-4 months. The reasons for reducing the certainty of evidence were inconsistency, imprecision, indirectness, or high risk of bias (Table 2).”

3. The results on lipid profile seems irrelevant to the aim of the study and these are already established, I would remove them.

**Answer:** Thank you for the suggestion. We have incorporated the lipid profile data as supplementary information. Please refer to 'Supplementary Table 6 for overall analysis and Supplementary Table 7 for treatment duration.

4. Please define “chronic diseases”, also, a subgroup analysis according to the primary disease seems important in current study.

**Answer:** We agree with the reviewer and appreciate the comment. In response, we have added a sentence describing the chronic diseases (lines 163-168) as follows: “Furthermore, the selection was restricted to studies involving adults with chronic diseases receiving statin treatment for a minimum of three months. The former is defined as conditions that last one year or longer and require ongoing medical treatment or limit activities of daily living [24] such as CVD, diabetes, hypertension, obesity, arthritis, Alzheimer’s disease, epilepsy, asthma, chronic obstructive pulmonary disease (COPD), HIV, TB, etc.”

However, a subgroup analysis of the primary diseases could not be performed due to the variability of chronic diseases in the studied populations, resulting in insufficient numbers of studies for each specific disease group.

**Reviewer #3**: The manuscript presents a systematic review and meta-analysis on the anti-inflammatory effects of statins in chronic diseases, evaluating various inflammatory markers and lipid profiles. While the research is extensive and methodologically sound, there are several areas that need significant improvement to enhance the clarity, rigor, and impact of the findings.

**Answer:** We thank the reviewers for this positive comment.

**Issues:**

**Introduction**:

1. The introduction is lengthy and contains redundant information. It should be more concise, focusing on the rationale and objectives of the study.

**Answer:** We thank the reviewer for this comment and have addressed these concerns in our response to reviewer 1, under point 1.

2. The clinical importance of the anti-inflammatory effects of statins needs to be more explicitly stated.

**Answer:** We thank the reviewer for this statement and have added the clinical importance at the end of the introduction and discussion as follows:

“The outcomes of this meta-analysis will feed into evidence-based protocols for statin therapy, addressing lipid abnormalities and mitigating inflammatory processes in disease progression. If proven effective, clinicians may consider the varying efficacy of statins in modulating inflammation in adults with chronic diseases.” Lines 115-119 in the revised manuscript.

“These findings have significant clinical implications for the management of chronic diseases characterized by inflammation, such as cardiovascular disease and chronic renal failure. Incorporating statin therapy into treatment protocols not only addresses lipid abnormalities but also potentially mitigates inflammatory processes implicated in disease progression. Clinicians may consider the differential efficacy of statins in inflammation modulation when tailoring treatment strategies for individual patients.” Lines 629-634 in the revised manuscript.

**Result:**

The high heterogeneity (I2 > 75%) in many analyses raises concerns about the comparability of the included studies. This should be more thoroughly addressed in both the results and discussion sections.

**Answer:** We thank the reviewer for raising this concern and have now extended the discussion and limitation section to include the following:

“We used random-effects models to account for variability among studies and provide more conservative estimates of the overall effect. Also, sensitivity analyses were performed to assess the robustness of the findings by excluding studies with skewed evidence, as shown in S9 Table. Despite these efforts, high heterogeneity remains a limitation that impacts the generalizability of the meta-analytic conclusion. To address publication bias, we employed comprehensive literature search strategies, including backward citation searching and trial registries. Additionally, we used statistical methods such as funnel plots and Egger’s test to detect and adjust for potential bias. However, these methods have limitations, and the possibility of residual bias cannot be entirely ruled out.” Lines 612-620 in the revised manuscript.

“The observed heterogeneity suggests that factors such as participant demographics, differences in study populations, baseline disease severity, treatment durations, and statin dosage might influence treatment outcomes.” Lines 491-493 in the revised manuscript.

**Discussion:**

1. The discussion is somewhat descriptive and needs to be more analytical. There should be a deeper exploration of why certain statins (e.g., atorvastatin) appear more effective than others.

Answer: We thank the reviewer for this comment and have now extended the discussion to include a deeper exploration of the differential effects of statins on inflammation, lines 623-629 in the revised manuscript, as follows: “The greater anti-inflammatory efficacy of rosuvastatin and atorvastatin is primarily attributed to their minimal first-pass effect and relatively slow hepatic elimination. In contrast, other statins such as pravastatin undergo extensive first-pass metabolism and is rapidly inactivated by the liver before reaching systemic circulation. As a result, atorvastatin and rosuvastatin have longer elimination half-lives of 14 and 19 hours, respectively, while pravastatin's elimination half-life is only 2 hours.

2. The implications of the findings for clinical practice should be discussed in more detail. How might these results influence treatment guidelines?

**Answer:** We thank the reviewer for this suggestion and have added the following in the discussion: ‘These findings have significant clinical implications for the management of chronic diseases characterized by inflammation, such as cardiovascular disease and chronic renal failure. Incorporating statin therapy into treatment protocols not only addresses lipid abnormalities but also potentially mitigates inflammatory processes implicated in disease progression. Clinicians may consider the differential efficacy of statins in inflammation modulation when tailoring treatment strategies for individual patients.’ Also see lines 629-634 in the revised manuscript.

3. The limitations section should be expanded. For example, the potential impact of publication bias and the high heterogeneity should be discussed more comprehensively.

**Answer:** We thank the reviewer for this comment. We have now expanded the limitation section into the discussion as highlighted below:

“An important limitation of this meta-analysis is that the lipid-lowering effect sizes of the various statins are derived from highly heterogeneous studies with variable baseline lipids and data on LDL-C and TC changes were not available for all statins. These results should thus not be interpreted as direct comparisons of statin potency. In this study, we used random-effects models to account for variability among studies and provide more conservative estimates of the overall effect. Also, sensitivity analyses were performed to assess the robustness of the findings by excluding studies with skewed evidence, as shown in S11 Table. Despite these efforts, high heterogeneity remains a limitation that impacts the generalizability of the meta-analytic conclusion. To address publication bias, we employed comprehensive literature search strategies, including backward citation searching and trial registries. Additionally, we used statistical methods such as funnel plots and Egger’s test to detect and adjust for potential bias. However, these methods have limitations, and the possibility of residual bias cannot be entirely ruled out.” Also, see lines 608-620 in the revised manuscript.
