# Supplementary material for: Impact of statins as immune-modulatory agents on inflammatory markers in adults with chronic diseases: A systematic review and meta-analysis
Source: PLoS One. 2025 May 29;20(5):e0323749. doi: 10.1371/journal.pone.0323749 (PMC12121830; doi:10.1371/journal.pone.0323749)
Supplement: Attachment — Submitted filename: Response_to_Reviewers_auresp_2.docx [file pone.0323749.s015.docx]

**Review Comments**

**1. The protocol mentioned a search in the ScienceDirect database, but the final manuscript does not reflect this. It would be beneficial to provide a rationale for deviating from the protocol in this regard.**

We appreciate the reviewer's comment. As specified in our published protocol (Sabeel and Motaung et al., BMJ Open 2020), we planned to conduct searches using PubMed (Medline), Scopus, Web of Science, and the Cochrane Library of Systematic Reviews. This is detailed in the published abstract, search strategy, and Figure 1. However, ScienceDirect was not included in our published protocol, and therefore, no searches were performed using this database.

**2. Reviewer 1 raised a question about the difference between the protocol and the manuscript, particularly regarding the inclusion of non-RCT studies. It is important to justify the inclusion of non-RCT studies and explain how they contributed to the findings**

Thank you for highlighting this point. We agree with the reviewer’s suggestion to justify the inclusion of non-randomized studies (NRS). Although our primary focus was on randomized controlled trials (RCTs), the significant number of high-quality NRS that evaluated the impact of statins on inflammatory markers prompted us to incorporate them into the systematic review. Including NRS for descriptive analysis has enriched our understanding of the immune-modulatory effects of statins, as demonstrated by the 41 NRS (87.2%) that reported a reduction in CRP levels. Furthermore, these NRS findings supported and complemented the data from RCTs, particularly in areas where RCT data were limited.

**3. I strongly recommend that the authors discuss the clinical application of statin use, including the magnitude of effect size (small, moderate, and large) and the minimum clinically important difference (MCID).**

Thank you for this valuable comment. The minimum clinically important difference (MCID) represents the smallest clinical improvement that is meaningful to the patient, incorporating both subjective and objective factors. To fully address this point, prospective studies are needed. The MCID often depends on the specific treatment indication. For example, the target for lowering cholesterol may require a different statin compared to when the statin is used to manage systemic inflammation.

The mean differences reported in our meta-analysis stem are derived from the heterogeneous characteristics of the included studies. These variations encompass a range of chronic diseases, diverse patient populations, different statin types, dosages, durations, and baseline variability. Additionally, we are not aware of published MCID for statins regarding overall chronic conditions. As a result, we are unable to interpret the results using the magnitudes of effect sizes or MCID. However, we have addressed the magnitude of effect size in our discussion, as follows (line 494 - 499): “Based on several assumptions, the effect size of statins on inflammatory markers in this meta-analysis was small to moderate and of limited clinical significance for statins overall. However, atorvastatin showed a likely large and clinically relevant impact on IL-6 and TNF-a, while fluvastatin had a very large effect on CRP, and rosuvastatin exhibited a moderate-to-large effect on CRP”

**4. The protocol mentioned a subgroup analysis based on statin dosage, but this was not reflected in the forest plot and table.**

Thank you for the feedback. We acknowledge that the protocol initially included a plan for subgroup analysis based on statin dosage. However, during the review process, we found an insufficient number of studies to create meaningful subgroup analyses. As a result, we were unable to perform this analysis.
