# Supplementary material for: Impact of statins as immune-modulatory agents on inflammatory markers in adults with chronic diseases: A systematic review and meta-analysis
Source: PLoS One. 2025 May 29;20(5):e0323749. doi: 10.1371/journal.pone.0323749 (PMC12121830; doi:10.1371/journal.pone.0323749)
Supplement: Attachment — Submitted filename: Response_to_Reviewers_auresp_3.docx [file pone.0323749.s016.docx]

**Review Comments**

**Reviewer #1:**

1. **The manuscript presents an engaging and well-composed exploration of an intriguing topic. However, several areas warrant attention for improvement. Firstly, the exclusion of cancer and malignancy patients, including those with HIV or Arthritis, raises questions.**

**Our response**: We appreciate the reviewer for the comment. We excluded participants with cancer or malignancy from the systematic review and meta-analysis because we considered cancer a potential confounding factor. Additionally, the variability in cancer types and the heterogeneity in stages within the same cancer type could introduce significant complexity to the findings. We have further acknowledged this exclusion as a limitation of our study and have accordingly included the above statement in the limitation section of the updated manuscript (lines 550-551). Patients with HIV and arthritis were included in our analysis as stated in our original manuscript in lines 136-139 of the manuscript.

2. **It would be beneficial for the authors to consider including all patient groups and to define a category for health status participants in the subgroup analysis. This could provide valuable insights into the effect of statins on inflammation among these participants.**

**Our response**: We agree with the reviewer. However, a subgroup analysis of the primary diseases could not be performed due to the variability of chronic diseases in the studied populations, resulting in insufficient numbers of studies for each specific disease group.

3. **Secondly, I would appreciate clarification regarding the GRADE assessment method employed.**

**Our response**: We thank the reviewer for raising this point. We have now clarified the GRADE assessment method which now can be read as follows: “The Grading of Recommendations, Assessment, Development, and Evaluations (GRADE) approach was used to rank the certainty of evidence as it related to the studies that contributed data to the meta-analyses for the prespecified outcomes. The GRADE tool includes five considerations (study limitations, inconsistencies of results, imprecision, indirectness, and publication bias) to assess the certainty of the evidence from RCTs that can be classified as high, moderate, low, or very low [19]. The decisions to downgrade the quality of evidence were justified using footnotes. One review author (KAN) made judgments about the evidence certainty, and a second review author (APK) checked this evaluation.” Lines 182-190, in the revised manuscript.

4. **Specifically, what approach was adopted to address heterogeneity?**

**Our response:** We thank the reviewer for this query. We explored the potential sources of heterogeneity using several approaches such as Random effect model, forest plots, I^2^ statistics, subgroup analyses, and Q-test based on the Analysis of Variance (ANOVA). These methods are detailed in lines 200-206 of the statistical analysis section.

5. **Was a null effect or small effect approach selected? This information appears to be missing.**

**Our response:** Thank the reviewer for raising this point. Indeed, the null effect was selected, and we have now added to the data analysis session a sentence: “The difference was statistically significant if P<0.05”. Lines 212-213.

6. **Thirdly, the rationale behind utilizing the GRADE assessment should be elaborated upon. The authors should integrate the GRADE results into their discussion of findings to enhance the manuscript's depth**.

**Our response**: Please view our response to point 2 above.

We have now incorporated the GRADE results into the discussion section, and added the following to the limitations session:

“Notably, the low to moderate-certainty of evidence, as assessed using GRADE, being attributable to imprecision, inconsistency or high risk of bias of some trials, suggests that further research may change these results.” Lines 501-503.

7. **Lastly, figures in the manuscript are of low quality and lack engagement. Improving these would significantly enhance the overall presentation of the work.**

**Our response**: We thank the reviewer for this comment. We now updated the figures with higher resolution.
